# Novel Flowchart Guiding the Non-Surgical and Surgical Management of Peri-Implant Complications: A Narrative Review

**DOI:** 10.3390/bioengineering11020118

**Published:** 2024-01-25

**Authors:** Takahiko Shiba, Keiji Komatsu, Yasuo Takeuchi, Tatsuro Koyanagi, Yoichi Taniguchi, Toru Takagi, Shogo Maekawa, Takahiko Nagai, Ryota Kobayashi, Shunsuke Matsumura, Sayaka Katagiri, Yuichi Izumi, Akira Aoki, Takanori Iwata

**Affiliations:** 1 Department of Periodontology, Graduate School of Medical and Dental Sciences, Tokyo Medical and Dental University, Tokyo 113-8549, Japankbryperi@tmd.ac.jp (R.K.); aoperi@tmd.ac.jp (A.A.);; 2 Department of Oral Medicine, Infection, and Immunity, Harvard School of Dental Medicine, Boston, MA 02115, USA; 3 Department of Lifetime Oral Health Care Science, Graduate School of Medical and Dental Sciences, Tokyo Medical and Dental University, Tokyo 113-8549, Japan; 4 Weintraub Center for Reconstructive Biotechnology, UCLA School of Dentistry, Los Angeles, CA 90095, USA; 5 Oral Care Periodontics Center, Southern TOHOKU Research Institute for Neuroscience, Southern TOHOKU General Hospital, Koriyama 963-8052, Japan

**Keywords:** peri-implantitis, peri-implant mucositis, dental implants, Er:YAG laser, free gingival graft, implantoplasty, connective tissue graft, keratinized mucosa, vestibular depth, cone-beam computed tomography

## Abstract

Peri-implant diseases, such as peri-implant mucositis and peri-implantitis, are induced by dysbiotic microbiota resulting in the inflammatory destruction of peri-implant tissue. Nonetheless, there has yet to be an established protocol for the treatment of these diseases in a predictable manner, although many clinicians and researchers have proposed various treatment modalities for their management. With the increase in the number of reports evaluating the efficacy of various treatment modalities and new materials, the use of multiple decontamination methods to clean infected implant surfaces is recommended; moreover, the use of hard tissue laser and/or air abrasion techniques may prove advantageous in the future. Limited evidence supports additional effects on clinical improvement in antimicrobial administration for treating peri-implantitis. Implantoplasty may be justified for decontaminating the implant surfaces in the supracrestal area. Surgical treatment is employed for advanced peri-implantitis, and appropriate surgical methods, such as resection therapy or combination therapy, should be selected based on bone defect configuration. This review presents recent clinical advances in debridement methods for contaminated implant surfaces and regenerative materials for treating peri-implant bone defects. It also proposes a new flowchart to guide the treatment decisions for peri-implant disease.

## 1. Introduction

In recent decades, the incidence of biological complications associated with dental implants has increased due to their increased use to replace missing or damaged teeth. Implant-related complications, specifically, can be divided into four main categories: biological, mechanical, and iatrogenic failures and inadequate adaptation. Biological failure can be further categorized as early failure due to unsuccessful osseointegration and late failure due to unsuccessful maintenance of the achieved osseointegration [1]. Early failure is mainly attributed to insufficient primary stability, surgical trauma, and the occurrence of infections. Conversely, late failure is more frequently associated with occlusal overload and peri-implantitis induced by poor oral hygiene [1]. Recently, titanium particles have been suggested as a possible cause of implant failure [2]. 

Peri-implant mucositis and peri-implantitis, categorized as late failures, are inflammatory conditions that are caused by bacterial plaque and affect the tissues around dental implants [3]. According to a recent meta-analysis, the prevalences of peri-implant mucositis and peri-implantitis were 42.9% and 21.7%, respectively [4]. These peri-implant diseases often cause rapid destruction of the tissues around an implant and require prompt and thorough therapeutic interventions [5], as treatment success depends on continual bacterial elimination from the contaminated implant surface and arrest of inflammatory processes [6]. Various mechanical and chemical decontamination methods have been suggested, either alone or in combination [7,8,9], for the elimination of bacteria from the micro- and macro-complex topography of implant surfaces. Although peri-implantitis is often treated using non-surgical treatment methods followed by surgical management, the success rate is low [10]; the non-surgical therapeutic approach alone is considered particularly inadequate in managing this condition [11]. During surgical treatment, soft tissue management (resection or grafting) is occasionally performed in conjunction with plaque debridement. Additionally, regenerative approaches are often used adjunctively to achieve bone regeneration and re-osseointegration around the implant, and various regenerative materials are currently being used for these purposes. The efficacy of surgical treatments for peri-implantitis has been evaluated in several studies, but factors such as the size and morphology of peri-implant bone defects, treatment procedures, and observation periods vary considerably across these studies, and comparing the effectiveness of each surgical treatment is challenging. Moreover, the results are often inconsistent even in studies with similar study designs. Currently, no clinical guidelines have been established for the treatment of peri-implant mucositis or peri-implantitis, yet the number of randomized controlled trials (RCTs) on these conditions has only been increasing. Therefore, reliable and practical clinical guidelines for the management of peri-implant mucositis and peri-implantitis are urgently required.

This narrative review summarizes the current knowledge regarding the diagnostic methods for evaluating the risk and status assessment of peri-implant diseases as well as decision-making protocols for their non-surgical and surgical treatments. We also propose a new flowchart to guide treatment decisions for peri-implant diseases and further recommend employing the clinical parameter of bleeding on probing (BOP) for initial screening of peri-implant status along with additional analyses such as bone defect configuration and keratinized mucosa (KM) thickness and width assessments. 

## 2. Methods

The Medline/PubMed and Google Scholar databases were searched for relevant articles on each addressed topic published until September 2023. All papers listed in the table are extracted from RCTs. The search strategy for RCT used for Medline/PubMed was as follows: ((“peri-implantitis”[All Fields] OR “peri-implant mucositis”[All Fields]) AND “non-surgical”[All Fields] AND “randomized controlled trial”[Publication Type]) AND (randomizedcontrolledtrial[Filter]) for papers about the treatment of peri-implant diseases using non-surgical therapy at August 2023 and (((“peri implantitis”[MeSH Terms] OR “peri implantitis”[All Fields] OR (“peri”[All Fields] AND “implantitis”[All Fields]) OR “peri implantitis”[All Fields]) AND (“surgical procedures, operative”[MeSH Terms] OR (“surgical”[All Fields] AND “procedures”[All Fields] AND “operative”[All Fields]) OR “operative surgical procedures”[All Fields] OR “surgical”[All Fields] OR “surgically”[All Fields] OR “surgicals”[All Fields]) AND (“surgical flaps”[MeSH Terms] OR (“surgical”[All Fields] AND “flaps”[All Fields]) OR “surgical flaps”[All Fields] OR “flap”[All Fields])) NOT (“review”[Publication Type] OR “review literature as topic”[MeSH Terms] OR “review”[All Fields])) AND (randomizedcontrolledtrial[Filter]) for papers about the treatment of peri-implant diseases using surgical therapy at September 2023. Papers that were not published in English or were not considered relevant based on the title and abstract were excluded. Full-text reviews were performed as the next step to further exclude articles that did not fit into the scope of this review.

## 3. Non-Surgical Treatment

### 3.1. Debridement Methods in Non-Surgical Treatment 

Non-surgical debridement has been widely employed for managing peri-implant mucositis and peri-implantitis. It is less invasive than surgical procedures and can result in reduced patient discomfort, shorter recovery periods, and lower incidences of post-treatment complications. In addition, non-surgical debridement allows for timely intervention, which may be preferable for both patients and clinicians and has proved effective for peri-implant mucositis [12]. Currently, manual instruments and ultrasonic devices are the main tools and devices employed for non-surgical mechanical debridement of contaminated implant surfaces [13,14,15,16,17]. Several RCTs have examined the efficacy of these tools and devices in managing peri-implant complications. These mechanical debridement methods have been shown to reduce peri-implant inflammation. Renvert et al. compared two non-surgical approaches (treated with either titanium manual instruments or ultrasonic devices) for managing peri-implantitis and observed a noticeable reduction in both the plaque and bleeding scores. However, no impact on the probing depth (PD) was observed [15]. In addition, no significant difference was observed in treatment outcomes between the manual and ultrasonic instruments groups. Thus, although both instruments are therapeutically effective to some extent, neither is superior in promoting healing. Furthermore, upon comparison between repetitive treatments using oscillating chitosan brushes and titanium curettes, significant reductions in the PD and bleeding index were observed in both the treatment groups after 6 and 12 months compared to those at baseline [18,19]. Thus, these studies support the opinion that non-surgical interventions, albeit limited, can indeed exert a positive influence in controlling peri-implant inflammation. 

The clinical effectiveness of ultrasonic devices and air abrasives in peri-implant therapy has been compared in several studies. Air-abrasive decontamination using agents such as amino acid glycine powder and erythritol was introduced for non-surgical decontamination of implant surfaces [20,21,22]. Hentenaar et al. demonstrated comparable effectiveness of both ultrasonic devices and air abrasives in the treatment of peri-implantitis in an RCT [21]. Similarly, studies comparing air abrasives and erbium-doped yttrium aluminum garnet (Er:YAG) laser debridement have shown similar clinical improvement [23,24]. However, the incorporation of air abrasives in conventional non-surgical techniques offers minimal additional benefit, thus limiting its clinical significance [20,25]. On the other hand, an RCT comparing air-abrasive and chemical disinfection using chlorhexidine (CHX) demonstrated a greater reduction in the BOP with air abrasive disinfection [26]. Although evidence suggests that air abrasives are beneficial in treating peri-implantitis, determining their superiority remains challenging. Thus, continuous assessment of the appropriate positioning and applicability of air abrasives amidst evolving treatment modalities is necessary.

Lasers are a reasonable treatment option when considering the intricate surface morphology of implant fixtures [27,28,29]. A study emphasized that laser decontamination is often not employed as a stand-alone application but rather as an adjunct to standard mechanical debridement, and suggested the supplementary advantages of diode lasers in non-surgical treatments to be small [27]. However, adjunctive beneficial effects of diode lasers have also been reported [29]. Another study reported that non-surgical mechanical debridement with adjunctive antimicrobial photodynamic therapy (aPDT) was as effective as adjunctive topical minocycline microspheres in reducing mucosal inflammation for up to 12 months [30]. Comparative research delineating the differences between various lasers is sparse, emphasizing the urgent need for clear guidelines for optimal laser selection and treatment protocols. The contents of this section are summarized in Table 1.

### 3.2. Effectiveness of Local and Systemic Antibiotics and Antiseptics in Non-Surgical Treatment

Administrations of various antibiotics, including amoxicillin, metronidazole, and azithromycin, have been investigated as supplementary treatments for the management of peri-implantitis in RCTs. However, reaching definitive conclusions regarding the overall effectiveness of systemic antibiotics in non-surgical treatment [31,32,33,34] is challenging. Moreover, Blanco et al. [35] reported beneficial effects of systemic administration of metronidazole in combination with non-surgical treatments. They observed significant improvements in the clinical parameters, such as PD, clinical attachment loss (CAL), and bone regeneration, after 12 months of treatment compared with those treated with placebo. Nevertheless, it is worth noting that the success rate of these interventions remained relatively low; only 56.3% of patients bore the success criteria. Although certain antibiotics such as metronidazole have demonstrated successful outcomes in specific cases, the use of antibiotics in peri-implantitis may be considered as part of individualized treatment approaches.

Daily use of antiseptics is a common plaque control measure for preventing periodontal and peri-implant diseases. A recent study by Alqutub et al. demonstrated the efficacy of post non-surgical treatment maintenance using CHX, NaCl, and herbal mouthwashes for short-term relief from peri-implant mucositis [36]. Another study also highlighted the potential complementary benefits of herbal-based and 0.12% CHX oral rinses to non-surgical mechanical debridement [37]. This is further supported by studies emphasizing the positive outcomes of integrating hypochlorite-based formulas with conventional treatments [38]. However, some reports could not successfully demonstrate the effectiveness of these antiseptics. A few studies have found limited to no additional benefit upon implementation of 0.12% CHX mouthwash in the treatment regimen [39]. Similarly, the additional application of CHX or chloramine gel after mechanical cleaning did not yield significant benefits [40,41]. Additionally, although the use of various mouth rinsing agents has demonstrated significant reductions in certain inflammatory markers, determining the type of mouthwash most appropriate and effective for peri-implantitis treatment remains challenging [42].

In summary, the use of antiseptic mouthwashes as an adjunct to non-surgical mechanical debridement may be helpful in suppressing peri-implant mucositis and peri-implantitis. However, considering the potential variability in the outcomes based on specific agents and individual patient conditions, a tailored and comprehensive approach is warranted. The contents of this section are summarized in Table 2.

### 3.3. Type of Materials Considered for the Non-Surgical Treatment of Peri-Implant Diseases

Some pioneering techniques for non-surgical management of peri-implantitis are gaining attention owing to the increasing demand for effective and minimally invasive approaches. The use of enamel matrix derivatives (EMD) for non-surgical treatment has emerged as a promising strategy [43]. Kashefimehr et al. highlighted the potential of EMD, particularly when combined with mechanical debridement therapy, for treating peri-implant mucositis. They conducted a double-blind clinical trial in patients with peri-implant mucositis and demonstrated that the adjunctive use of an EMD with mechanical debridement led to significant improvements in BOP and PD after three months, compared to the debridement alone. Furthermore, inflammation was significantly curtailed by EMD application, as evidenced by the reduced levels of interleukin (IL)-6 and IL-17 in the peri-implant crevicular fluid [43]. 

Furthermore, the use of probiotics is emerging as an intriguing strategy for the management of peri-implant diseases [44,45]. In one RCT, the administration of *Lactobacillus reuteri* as an oral probiotic along with non-surgical mechanical therapy resulted in significant improvements in the clinical parameters of implants with peri-implant mucositis or peri-implantitis. Specifically, improvements were observed in the patient-wide BOP and PD values at the implant sites over a 90-day period [44]. However, this approach had limited effect on the microbiota in peri-implant tissue; the only parameter that showed a significant decrease was the *Porphyromonas gingivalis* count in peri-implant mucositis. Thus, the microbiological effects seemed limited despite the presence of immediate benefits of this combination therapy. Studies investigating such innovative treatment approaches may change non-surgical interventions for peri-implant diseases. The contents of this section are summarized in Table 3.

## 4. Surgical Treatment

### 4.1. Decontamination Methods of the Implant Surface for Surgical Treatment of Peri-Implantitis

Surgical approaches have been recommended for treating peri-implantitis [46]. However, the improvement rate of peri-implantitis after surgical debridement with conventional surgical procedures employed in periodontitis remains challenging, as less than 50% of affected implants can be recovered after surgical treatment in most cases [17,47]. Various therapeutic approaches have been used to enhance the efficacy of surgical treatments for peri-implantitis.

Although implant surface decontamination methods, such as Er:YAG laser, plastic curettes, cotton pellets, and gauze, have been compared, the results have been inconsistent. Some studies have demonstrated no superiority for any specific debridement technique [48,49,50,51,52]. In contrast, several RCTs have reported that certain debridement methods are more effective than others [53,54,55,56]. Pranno et al. evaluated the efficiency of bacterial removal from contaminated implant surfaces in patients with severe peri-implantitis by using mechanical debridement with air-powder abrasion, chemical decontamination with hydrogen peroxide and CHX gluconate, or a combination of mechanical-chemical decontamination. The amount of residual bacteria on the implant surfaces after mechanical debridement alone or combined mechanical-chemical decontamination was significantly lower than that after untreated or chemical decontamination alone, indicating the superiority of mechanical debridement to chemical decontamination [53]. Bombeccari et al. reported that the aPDT after the decontamination with plastic scalers and irrigation with a 0.2% CHX digluconate solution for 1 min in open flap debridement (OFD) significantly reduced the proinflammatory index of peri-implantitis at 24 weeks of follow-up compared to that of the control group [54] Regarding the difference in the mechanical debridement methods, the adjunctive use of Er:YAG laser in regenerative therapy significantly improved the PD compared to the control group; however, no significant difference was observed in terms of improvement in radiographic findings [55]. Romeo et al. compared the clinical outcomes with and without modification of the surface topography of the contaminated implant surfaces (implantoplasty) in the surgical treatment of peri-implantitis. They demonstrated that additional implantoplasty at the supra-crestal area as a means of decontamination during resective therapy could improve the PD, probing attachment level, and modified bleeding index compared to those of the control group at 24 months; however, the recession index in the control group was significantly lower than that in the experimental group. The cumulative survival rate of the implants in the implantoplasty with resective surgery group in this study was found to be 100% after 3 years [56]. 

At present, it is difficult to conclude which debridement method is superior; furthermore, complete decontamination of the infected implant surface is challenging even following application of these methods. Multiple decontamination methods have been recommended for cleaning peri-implant surfaces. The contents of this section are summarized in Table 4. 

### 4.2. Use of Antibacterial Agents in Surgical Treatment 

Some randomized control studies have suggested that adjunctive local and systemic antibiotic and antiseptic administration during OFD for peri-implantitis did not provide clinical benefits compared to OFD alone [57,58]. Teughels et al. reported the efficacy of choline-stabilized orthosilicic acid (CS-OSA) in patients who underwent OFD for the treatment of peri-implantitis. CS-OSA has been known to stimulate bone collagen formation in osteopenia, and oral administration of CS-OSA twice daily for one year prevented further bone loss after OFD for peri-implantitis in a patient [59]. Limited evidence exists regarding the use of antibacterial agents and decontamination methods during surgical treatment of peri-implantitis, thus warranting further research. The contents of this section are summarized in Table 5. 

### 4.3. Types of Regeneration Material Used in Surgical Treatment of Peri-Implantitis 

Many RCTs have compared the efficacy of various regenerative materials and their combinations in surgical procedures for treating peri-implantitis [60,61,62,63,64,65,66,67,68,69]. A few studies have reported no improvement in the clinical parameters upon the use of regenerative materials in peri-implant surgical treatments [60,61]. Wohlfahrt et al. performed OFD with or without porous titanium granules (PTGs) and compared the 12-month post-treatment outcomes [60]. They showed that both the surgical interventions induced a reduction in the PD and biomarker levels involved in extracellular matrix degradation, regulation of bone resorption, and bone formation ( i.e., matrix metalloproteinase (MMP)-8, IL-6, insulin, and osteoprotegerin) in the peri-implant sulcal fluid; however, no significant differences were observed between the two groups. Isehed et al. also showed no additional improvement in the bone level or PD, even when EMD was used in combination with OFD [61]. However, some RCTs have shown that the combination of OFD and regenerative materials is more effective than OFD alone [62,63,64,65]. Derks et al. reported that OFD with cancellous bone granules plus 10% highly purified porcine collagen demonstrated statistically less buccal gingival recession than OFD alone, although OFD with graft material did not improve the reduction in the PD or BOP at 12 months after surgery [62]. Jepsen et al. also reported that significantly enhanced radiographic defect filling was observed in the treatment of OFD with PTGs compared to that with OFD alone, even though no significant differences were observed in the PD and BOP between the two groups 12 months after surgery [63]. Another comparative study examining OFD with and without PTGs reported similar results [64]. Emanuel et al. administrated D-PLEX500 (a biodegradable prolonged release local doxycycline formulated with β-tricalcium phosphate bone graft) in the surgical treatment of peri-implantitis. At 12 months after surgery, the D-PLEX500 group showed more significant improvements in clinical parameters such as PD, CAL, radiographic bone levels, and BOP than the OFD-alone group; however, there was no significant difference in CAL between groups [65]. As for the improvement in the soft tissue morphology around implants, Solonko et al. compared the efficacy of an apically positioned flap with an autologous free gingival graft (FGG) or a collagen matrix. Both treatments resulted in a significant increase in peri-implant KM at 12 months after treatment, whereas the increase in the KM was significantly greater with FGG. No significant differences were observed in the PD, BOP, or vestibular depth (VD) in the study [66]. A few comparative randomized controlled studies employed regenerative substitutes in both the test and control groups [67,68,69]. The application of a natural bone mineral in combination with a collagen membrane (NBM + CM) showed higher mean PPD reduction than nanocrystalline hydroxyapatite (NHA) (NBM + CM: 2.4 ± 0.8 mm vs. NHA: 1.5 ± 0.6 mm at 24 months after surgery and NBM + CM: 2.5 ± 0.9 mm vs. NHA: 1.1 ± 0.3 mm at 48 months after surgery) and CAL gains (NBM + CM: 2.0 ± 0.8 mm vs. NHA: 1.0 ± 0.4 mm at 24 months after surgery and NBM + CM: 2.0 ± 1.0 mm vs. NHA: 0.6 ± 0.5 mm at 48 months after surgery), indicating the application of NBM + CM to have superior clinical outcomes compared to those with NHA; however, statistical analysis was not performed in these studies [67,68]. Schwarz et al. evaluated the efficacy of NHA application and a bovine-derived xenograft combined with a collagen membrane (BDX + CM) in the surgical treatment of peri-implantitis. No significant difference was observed in the PD and CAL between the two groups 6 months after surgery owing to the significant decrease in the PD and increase in the CAL in both groups [69]. 

At present, only a limited number of RCTs have been conducted on regenerative treatment of peri-implantitis; thus, determining which material is superior is challenging. Radiographic improvement of the bone would indeed be further enhanced with the use of regenerative materials. However, we should cautiously interpret whether radiographic improvement actually leads to clinical improvement of the peri-implant condition [67]. The contents of this section are summarized in Table 6.

## 5. Clinical Parameters for Evaluating the Peri-Implant Condition

### 5.1. Probing Depth

The depth of the peri-implant sulcus in healthy implants is generally 3–4 mm [70], whereas diseased implant sites (peri-implant mucositis and peri-implantitis) are generally slightly deeper PD (4–6 mm) [71], and a 6 mm PD has been suggested as the threshold for progressive bone loss [72]. The discrepancy between the anatomical peri-implant sulcus and PD is slight in the absence of inflammatory changes but increases with the exacerbation of inflammation around the teeth [73]. As with natural teeth, the tip of periodontal probe can identify the apical border of the epithelium barrier with an error of approximately 0.2 mm for both healthy and peri-implant mucositis sites. However, at the sites of peri-implantitis, the measurement error is much greater at 1.5 mm [74]. Additionally, Serino et al. reported a discrepancy in the PD with or without a superstructure at the implants, and PD after prosthesis removal is highly correlated with the bone level of implants assessed at surgery [75]. Clinicians should also be aware of the fact that the presence or form of the implant superstructure can interfere with the PD measurements. Peri-implantitis sites exhibit increased PD and clinical signs of inflammation compared to those at baseline [76]. In cases where patients can receive maintenance care at the clinic where their implants were placed, repeated assessment of PD is very useful for evaluating the peri-implant condition. Conversely, a diagnosis of peri-implantitis can be considered in cases of a PD of ≥6 mm when information about the baseline PD is not available [3].

### 5.2. Bleeding on Probing

BOP, which is a sign of inflammation of the periodontal sulcus, is considered a valid predictor of future loss of attachment around teeth [77]. Moreover, the absence of BOP is a reliable indicator of periodontal tissue stability [78]. An examination of the BOP to monitor attachment loss around dental implants would be superior to that of natural teeth when a probing force of 0.25 N is used [79]. A meta-analysis revealed that the incidence of peri-implantitis in implants with BOP was 24.1%, with high variability among studies [80]. Although the positive rate of BOP seems to be low, it is considered reasonable because peri-implant mucositis is also included in BOP-positive implants. BOP with additional microbiological tests on natural teeth and implants significantly enhanced the diagnostic accuracy for attachment loss compared with BOP alone [79]. BOP is a reliable indicator of peri-implant inflammatory complications, although it should be used in combination with other parameters for the diagnosis of peri-implant diseases.

### 5.3. Radiographic Interpretation

Regular inspection of the marginal bone level around the implant site is important for maintaining implant health and early detection of peri-implant lesions. An annual marginal bone loss of less than 0.2 mm one year after implant placement has been regarded as one of the criteria for implant success [81]. Orthopantomograms and periapical radiographs are convenient and conventionally used as low-dose radiation tools to assess marginal bone loss around implants. On the other hand, visualizing buccal and lingual bone resorption using this method is challenging. Additionally, changes in the crestal bone morphology may only be noticeable when the resorptive lesions are of significant size and shape [82]. Serino et al. reported that the measurement of peri-implant bone loss based on periapical radiographs differed from that of clinical bone loss during surgery [83]. Accurate assessment of the extent [84] and configuration of the bone defect around the implant is important during decision making, such as whether regenerative therapy could be possible, in the treatment for peri-implantitis [85]. The recent advances in three-dimensional (3D) imaging using cone-beam computed tomography (CBCT) are remarkable, and the size and types of periodontal bone defects can be easily predicted using this technique [86,87]. However, X-ray artifacts can seriously decrease the quality of CBCT images [88] and sometimes make it impossible to assess bone resorption around dental implants. In addition, the quality of CBCT images is affected by various factors, including the device, field of view, X-ray beam quality, and image reconstruction parameters [89]. Color flow ultrasonography is a recently introduced non-invasive method for instantaneous assessment of dynamic tissue performance and degree of clinical inflammation at the implant site [90]. The combination of radiographic interpretation and ultrasound could facilitate observing changes in the marginal bone and determining bone defect configurations around implants in the future.

### 5.4. Bone Sounding

Christiaens et al. measured the bone levels at periodontal disease sites using clinical measurements, radiographs (analog and digital intraoral radiographs), and bone sounding (BS) without a flap elevation, and compared these values with the true bone levels measured when the flap was elevated [91]. BS was the most accurate, with all the other evaluation methods underestimating the true bone level [91]. Shan et al. compared the bone levels around the teeth among conventional methods using a 3D radiographic technique, BS, and direct measurements under open-flapped conditions. They showed high agreement between the BS measurements and true bone level, whereas the results of the 3D radiographic technique were weakly correlated [92]. Artifacts cannot be avoided when using CBCT and X-ray imaging. Many studies have used BS to determine the actual bone defect morphology around implants [50,93,94,95]; although BS is somewhat invasive, it shows high predictability of the peri-implant bone levels.

Using multiple clinical parameters is necessary for diagnosing peri-implant mucositis and peri-implantitis. Among the clinical parameters, BOP can be considered a useful tool for differentiating between healthy and diseased states. The removal of the superstructure is recommended for accurate PD measurements. BS should be used to select appropriate treatment methods when identification of the bone defects on radiographic images is challenging.

## 6. Management of Peri-Implant Health and Treatment Protocol of Peri-Implant Disease

**Figure 1:**
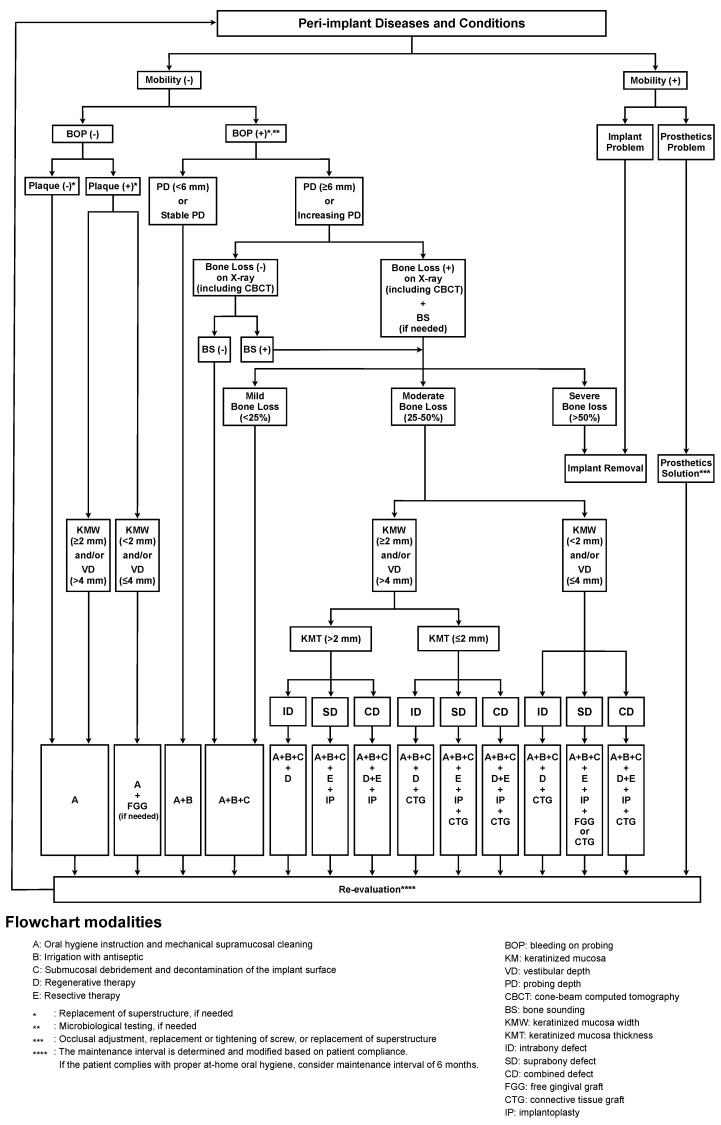
Proposed flowchart for management of peri-implant diseases (FMPD). The bleeding on probing (BOP) is used as an initial examination parameter in this protocol, except for mobility. Treatment options cumulatively increase depending on the severity of the peri-implant disease. An improperly shaped suprastructure that accelerates plaque accumulation should be converted into an appropriate suprastructure. The adjunct use of microbiological tests is sometimes recommended for the assessment of the peri-implant conditions when BOP is observed around the dental implant. If bone defects and their configuration cannot be evaluated by radiographic examination alone, bone sounding (BS) may be additionally recommended to accurately analyze the bone defect. Moreover, regular reexamination is required to evaluate treatment efficacy. This flowchart can be used at every patient visit, including a follow-up visit. The recommended interval for reexamination is at least once every 6 months. Implants with loss of osseointegration must be promptly removed.

It is now clear that peri-implant disease is more complex and challenging to treat than diseases involving natural teeth. Therefore, management of the health of the tissues surrounding functioning dental implants is essential. We propose a new management flowchart for peri-implant health and disease (Figure 1). The treatment plan for peri-implant disease is decided based on the mobility, BOP, presence of dental plaque, width of the KM and VD, PD, presence of bone loss, amount of bone defect configuration, and thickness of the KM. The BOP is considered the initial examination parameter to assess the peri-implant health condition, except for mobility, in the process of clinical screening. Microbiological tests as additional evaluation items may be helpful in assessing the risk of peri-implant diseases when BOP is observed in the soft tissue around the dental implant [79]. In addition, several clinical parameters, such as PD, plaque deposition, and radiographic examination, should be evaluated. Complete understanding of the peri-implant conditions is critical for planning treatments and supportive procedures. BS is sometimes useful when the morphology of the bone defect is not clearly understood on radiography or CBCT [91]. In addition, it is important to evaluate the KM and VD widths, KM thickness, and bone defect configuration to determine the appropriate treatment strategies for advanced peri-implant disease.

Similar to natural teeth, the need for a minimum amount of KM to maintain peri-implant tissue health remains controversial [96,97,98,99,100]. Some studies have suggested that implants with a reduced KM width of <2 mm are more susceptible to plaque accumulation and marginal inflammation [101,102,103,104]. The thickness of the preoperative gingival tissue also appears to affect the changes in the marginal bone around the implant. The crestal bone loss 1 year after implant placement in the sites with KM thickness ≤2.0 mm is less than in the sites with thickness >2.0 mm [105]. Halperin–Sternfeld et al. reported that sites with a shallow VD around dental implants presented with a lower KM width than sites with adequate VD in a 6-year retrospective longitudinal study [106]. Sites with a shallow VD (≤4 mm) around dental implants are associated with higher mucosal recession, increased relative attachment loss, and enhanced relative bone loss compared to sites with an adequate VD (>4 mm) [106]. Thus, minimum levels of KM and VD may be desirable to prevent bone resorption and maintain the soft tissue around the implant.

The surgical modality performed to improve the residual pocket after non-surgical treatment is also determined by the configuration of the bone defect. Schwarz et al. characterized the bone-defect configuration and distinguished between Class I (infrabony) and Class II (suprabony) defects in the crestal implant insertion area, and further subdivided Class I into five different configurations. Circular bone resorption (Class Ie) was most frequently observed at peri-implantitis sites [107]. Resective therapy has been proposed for Class II bone resorption because improving deficient bone conditions is challenging even with the application of regenerative therapy. In contrast, in Class I defects, circular bone resorption, such as Class Ie, has great potential for regeneration [85]. A systematic review by Daugela et al. assessed the efficacy of regeneration therapy for peri-implantitis and reported that the average radiographic marginal bone level fill was 1.97 mm, and the PD and BOP were reduced by 2.78 mm and 52.5%, respectively [108]; therefore, regenerative therapy can be recommended in cases of Class I bone resorption.

Combined defects (Class I and Class II) are often observed in daily clinical practice, and both regenerative and resective therapies may be required simultaneously or in stages. In cases of peri-implantitis with buccal deficiency and/or supracrestal and intrabony defects, combined surgical treatment with implantoplasty using a peri-implant regenerative approach is effective [48,109,110]. The combination of surgical treatment of peri-implantitis and soft tissue grafting might also be effective in controlling severe peri-implantitis lesions [111].

The major treatment elements in our flowchart are as follows: (A) oral hygiene instruction and mechanical cleaning; (B) irrigation with antiseptics; (C) debridement at the submucosal area and decontamination at the implant surface; (D) regenerative therapy; and (E) resective therapy. Treatment options increase cumulatively depending on the disease severity, similar to cumulative interceptive supportive therapy [112]. If plaque deposits are always observed at a site with inadequate widths of the KM and VD (<2 mm and ≤4 mm, respectively), protocol A plus FGG may be recommended to establish a cleanable oral environment. If the implant is diagnosed as peri-implant mucositis and peri-implantitis with mild bone loss (<25%), non-surgical treatments (protocols A, B, and C) are recommended [113,114]. For debridement and decontamination (protocol C), simultaneous use of multiple devices (such as ultrasonic scalers, titanium curettes, plastic curettes, Er:YAG laser, diode laser, air abrasion, etc.) is recommended as much as possible. In the case of moderate bone loss (25–50%) around an implant, surgical therapies (protocols D and E) can be applied based on the configuration of the bone defect. If the width of the KM and VD (<2 mm and ≤4 mm, respectively) and the thickness of the KM are inadequate (≤2 mm) in the case of moderate bone loss around an implant, surgical therapy comprising flap elevation with FGG or connective tissue graft is recommended [66,114]. For implants with severe bone loss (>50%) or mobility owing to implant fracture, the removal is recommended [114,115]. When retreatment with new implants is required after implant removal, transmucosal implants with a convergent hyperbolic design might be recommended based on a result of complication-free stability of marginal bone loss and BOP for up to 3 years [116]. Additionally, convergent collar implants with crowns fabricated using the biologically oriented preparation technique (BOPT) showed a significant increase in soft tissue volume at the papillae and the buccal margin around implants [117]. Finally, patients who were enrolled in a recall system and maintained a high standard of oral hygiene were reported to be able to keep a stable peri-implant condition for at least 5 years following peri-implant surgery [118].

It must be noted that this review did have some limitations. It only included articles published in English; hence, some pertinent articles published in other languages may have been excluded. Some relevant studies could have been overlooked because only titles and abstracts were used for screening. In addition, meta-analysis was not available because the experimental methods were not homogeneous in RCTs.

## 7. Conclusions

BOP is considered a useful screening parameter for signs of inflammation. Therefore, we propose that BOP should be the first parameter assessed after mobility evaluation for successful management of peri-implant health. If signs of inflammation or tissue destruction are observed, the condition of the peri-implant tissue must be carefully evaluated for treatment planning. Non-surgical treatment using multiple decontamination methods should be used initially to improve the diseased peri-implant condition. For advanced lesions, surgical treatment modalities should be considered based on the bone defect configuration and the condition of surrounding soft tissues. Surgical procedures, including those that involve the use of regenerative materials, should be considered in cases of infrabony defects. The newly proposed flowchart serves as an excellent and reproducible guideline for peri-implant health management and treatment planning for peri-implant diseases.

## Figures and Tables

**Table 1:** List of randomized control studies comparing non-surgical debridement methods.

Reference Number	Authors	Number of Patients	Intervention	Observation Period	Main Findings
[13]	Iorio-Siciliano et al. (2023)	54	Subgingival debridement of soft tissue-level and bone-level implants affected by peri-implant mucositis using a sonic scaler with a plastic tip.	6 months	Although non-surgical mechanical treatment improved PD and BOP for both tissue-level and bone-level implants, complete resolution of the disease could not be achieved.
[14]	Persson GR et al. (2010)	37	Mechanical debridement of peri-implantitis lesions using curettes or an ultrasonic device.	6 months	Both methods were ineffective in achieving consistent reductions in bacterial counts.
[15]	Renvert S et al. (2009)	37	Non-surgical, mechanical debridement for peri-implantitis using a titanium hand-instrument or an ultrasonic device.	6 months	Both treatments had comparable levels of improvements in plaque and bleeding scores. Neither approach was found to have any effect on PD.
[16]	Romandini M et al. (2022)	42	Control group: supra- and sub-marginal treatment using an ultrasonic instrument and titanium curettes 6 weeks before peri-implantitis surgery; test group: only supra-marginal cleaning 6 weeks before peri-implantitis surgery.	12 months	Submarginal instrumentation 6 weeks prior to surgical treatment did not have any additional benefit on peri-implantitis.
[17]	Wagner TP et al. (2021)	45	Non-surgical and surgical debridement for peri-implantitis using a Teflon curette and/or a stainless-steel Mini-Five curette.	12 months	Both types of debridement resulted in similar clinical outcomes.
[18]	Khan SN et al. (2022)	39	Debridement for peri-implantitis using an oscillating chitosan brush or titanium curette at baseline and 3 months later.	6 months	There were no discernible discrepancies in treatment outcomes between the two groups. The level of predictability of disease eradication was low.
[19]	Khan SN et al. (2023)	39	Non-surgical treatment of peri-implantitis using an oscillating chitosan brush or titanium curette every 3 months.	12 months	Clinical improvement was observed in both groups, although inflammation persisted in certain cases.
[20]	Ji YJ et al. (2014)	24	Peri-implant mucositis treatment using non-surgical debridement with and without glycine powder air-polishing.	3 months	Clinical parameters improved in both groups, but the use of glycine powder air-polishing did not provide any significant benefits as compared to mechanical debridement alone.
[21]	Hentenaar DFM et al. (2021)	79	Non-surgical peri-implantitis treatment using erythritol air polishing or piezoelectric ultrasonic scaling.	12 months	Neither treatment could resolve peri-implantitis effectively.
[22]	Riben-Grundstrom C et al. (2015)	37	Peri-implant mucositis treatment using glycine powder air-polishing or ultrasonic treatment at baseline and subsequently at 3-month and 6-month intervals.	12 months	The number of diseased sites was significantly lower in both treatment groups.
[23]	Person GR et al. (2011)	42	Peri-implantitis treatment using Er:YAG laser irradiation and air-abrasion.	6 months	Both methods failed to reduce bacterial counts, and clinical improvement was limited.
[24]	Renvert S et al. (2011)	42	Peri-implantitis treatment using Er:YAG laser or an air-abrasive device and a hydrophobic powder.	6 months	Both approaches resulted in a reduction in PD, the frequency of suppuration, and bleeding at implant sites.
[25]	Selimović A et al. (2023)	43	Low-abrasion erythritol air polishing as an adjunct to conventional non-surgical management of mild to severe peri-implantitis using titanium curettes and an ultrasonic device with a titanium tip.	12 months	The use of the erythritol air-polishing and conventional non-surgical treatment was unlikely to offer any additional clinical benefits.
[26]	Sahm N et al. (2011)	32	Non-surgical treatment of peri-implantitis using an air-abrasion device or mechanical debridement.	6 months	Both treatments showed similar CAL gains. However, glycine powder abrasion resulted in greater BOP reduction compared to carbon curette and chlorhexidine therapy.
[27]	Alpaslan Yayli NZ et al. (2022)	54	Conventional non-surgical mechanical treatment alone or mechanical treatment along with the additional 940 nm diode laser or 2780 nm Er,Cr:YSGG laser treatment for peri-implantitis.	6 months	Additional diode laser treatment with non-surgical mechanical treatment did not provide any additional benefits in terms of treatment outcome. However, Er,Cr:YSGG laser treatment was more efficient than the other two methods at the clinical and molecular levels.
[28]	Roccuzzo A et al. (2022)	30	Mechanical debridement for peri-implantitis with or without diode laser treatment on days 0, 7, and 14.	6 months	Diode laser treatment alongside non-surgical treatment did not offer significant advantages over mechanical treatment alone.
[29]	Tenore G et al. (2020)	30	Mechanical debridement therapy with/without diode laser treatment.	3 months	The adjunct use of the diode laser resulted in greater improvements in PD and BOP than conventional non-surgical treatment alone.
[30]	Bassetti M et al. (2014)	40	Non-surgical treatment after mechanical debridement for initial peri-implantitis: adjunctive local drug delivery or PDT.	12 months	Both adjunctive PDT and the adjunctive delivery of minocycline microspheres had a similar effect on reducing mucosal inflammation.

PD: probing depth, CAL: clinical attachment loss; BOP: bleeding on probing, Er:YAG: erbium-doped yttrium aluminum garnet laser, PDT: photodynamic therapy.

**Table 2:** List of randomized control studies on non-surgical treatment using local and systemic antibiotics, rinses, and gels.

Reference Number	Authors	Number of Patients	Intervention	Observation Period	Main Findings
[31]	De Waal YCM et al. (2021)	62	Mechanical debridement and decontamination with chlorhexidine for peri-implantitis. Test group patients also received systemic antibiotic therapy (AMX and MTZ).	3 months	Systemic administration of AMX and MTZ did not provide any additional improvement in clinical and microbiological outcomes compared to non-surgical treatment alone.
[32]	Hallström H et al. (2012)	48	Non-surgical debridement for peri-implant mucositis with or without systemic azithromycin administration for 4 days.	6 months	A decrease in implant bleeding over was observed in both groups, but there were no other significant differences between the groups.
[33]	Polymeri, A. et al. (2022)	37	Adjunctive systemic AMX and MTZ administration in patients undergoing non-surgical treatment of peri-implantitis.	3 months	Both approaches proved ineffective in completely resolving the inflammation around dental implants.
[34]	Shibli JA et al. (2019)	40	Non-surgical treatment of severe peri-implantitis with or without MTZ and AMX administration.	12 months	AMX and MTZ administration did not result in any additional benefits with non-surgical treatment.
[35]	Blanco C. et al. (2022)	32	Non-surgical treatment of peri-implantitis with or without adjunctive systemic MTZ administration.	12 months	Administering MTZ systemically as an adjunct to non-surgical peri-implantitis treatment was significantly beneficial.
[36]	Alqutub M. N et al. (2023)	60	Non-surgical mechanical debridement for the treatment of peri-implant mucositis followed by CHX treatment, 2% saline rinses, or herbal mouthwash use.	3 months	Use of CHX, herbal mouthwash, and saline rinse after non-surgical treatment was beneficial for short-term peri-implant mucositis management.
[37]	Alzoman H et al. (2020)	48	Non-surgical mechanical debridement for peri-implant mucositis with herbal or 0.12% CHX oral rinses.	3 months	Use of herbal and 0.12% CHX oral rinses led to significant reductions in peri-implant plaque index, BOP, and PD.
[38]	Cosola S. et al. (2022)	40	Non-surgical treatment of peri-implant mucositis using a CHX toothpaste and mouthwash or a hypochlorite-based brushing solution.	90 days	Combined use of the hypochlorite-based formula may help resolve peri-implant mucositis.
[39]	Menezes KM et al. (2016)	51	Non-surgical treatment of peri-implant mucositis using a 0.12% CHX mouthwash.	6 months	No additional benefit of the 0.12% CHX mouthwash was observed.
[40]	Heitz-Mavfield LJ et al. (2011)	29	Non-surgical mechanical debridement for peri-implant mucositis and brushing around the implant twice daily using a CHX or placebo gel.	3 months	Adjunctive CHX gel application did not enhance the results of mechanical debridement alone.
[41]	Roos-Jansåker AM et al. (2017)	18	Non-surgical treatment of peri-implantitis with/without local applications of a chloramine gel.	3 months	Both treatments effectively reduced mucosal inflammation, with no significant difference between the groups.
[42]	Philip J et al. (2020)	89	Non-surgical mechanical debridement and rinsing with either DEL, CHX, or a placebo for peri-implant mucositis.	3 months	All treatments reduced peri-implant mucositis symptoms, with no significant differences between the DEL, CHX, and placebo groups, except for a difference in plaque index between the CHX and placebo groups at 1 month.

AMX: amoxicillin, MTZ: metronidazole, CHX: chlorhexidine, NaCl: sodium chloride, DEL: delmopinol hydrochloride.

**Table 3:** Innovative approaches to non-surgical treatment of peri-implant diseases.

Reference Number	Authors	Number of Patients	Intervention	Observation Period	Main Findings
[43]	Kashefimehr A et al. (2017)	41	Mechanical debridement alone or with an enamel matrix derivative for peri-implant mucositis treatment.	3 months	The application of the enamel matrix derivative significantly improved clinical parameters and reduced cytokine levels compared to debridement alone.
[44]	Galofré M et al. (2018)	44	Patients with peri-implant mucositis or peri-implantitis received mechanical debridement combined with either daily probiotic or placebo administration for 30 days.	90 days	The probiotic improved the clinical parameters of affected implants; however, the impact on the peri-implant microbiota was limited.
[45]	Laleman I et al. (2020)	23	Non-surgical therapy for peri-implantitis with or without additional administration of *Lactobacillus reuteri* probiotics.	6 months	*L. reuteri* probiotics did not have any additional benefits.

**Table 4:** List of randomized control studies comparing debridement methods during surgical therapy.

Reference Number	Study	Number of Patients	Intervention	Observation Period	Main Findings
[48]	Schwarz F et al. (2011)	32	In the test group, implantoplasty at buccally and supracrestally exposed parts of implants and debridement of other implant surfaces using Er:YAG laser were conducted in combination with surgical therapy. In the control group, plastic curettes, cotton pellets, and sterile saline were used for the debridement of the implant surface.	24 months	There were no statistically significantly differences in clinical parameters between the Er:YAG laser and control groups.
[49]	Schwarz F et al. (2012)	24	The same as in the study by Schwarz et al. in 2011.	24 months	There were no significant differences in mean BOP and CAL values between the Er:YAG laser and control groups.
[50]	Schwarz F et al. (2013)	17	The same as in the study by Schwarz et al. in 2011.	48 months	The differences between the two surface decontamination methods did not influence the assessed long-term clinical outcome.
[51]	Papadopoulos CA et al. (2015)	19	Adjunctive diode laser irradiation with OFD for peri-implantitis. In the control group, debridement was performed using a plastic curette and a sterile saline-soaked gauze. Patients in the test group also underwent low-power diode laser irradiation (980 nm, 0.8 W) three times.	6 months	No clinical benefit of additional diode laser irradiation was observed.
[52]	Esposito M et al. (2013)	80	OFD for peri-implantitis; patients in the test group underwent debridement using curettes in addition to light-activated disinfection treatment.	12 months	The additional light-activated disinfection therapy did not yield any clinical improvements compared to mechanical cleaning alone.
[53]	Pranno N et al. (2021)	20	Surface decontamination for treating severe peri-implantitis: mechanical debridement with air-powder polishing, chemical decontamination with hydrogen peroxide and chlorhexidine gluconate, or a combination of mechanical and chemical decontamination. The implant was then carefully removed, and the amount of residual plaque on the surface was assessed.	12 months	Mechanical debridement with sodium bicarbonate and glycine powder was significantly superior to chemical decontamination with hydrogen peroxide and chlorhexidine gluconate in removing bacterial biofilm from infected implant surfaces.
[54]	Bombeccari GP (2013)	40	OFD with photodynamic therapy and debridement using plastic scalers.	6 months	A significantly lower proinflammatory index of peri-implantitis was observed in the photodynamic therapy group.
[55]	Wang CW et al. (2021)	24	Additional laser debridement during regenerative therapy for peri-implantitis.	6 months	There was a significantly greater reduction in PD at the site level in the test group than in the control group.
[56]	Romeo E et al. (2005)	17	In test group the surface was polished using a diamond bur, Arkansas burs, and silicone polishers in addition to the debridement using hand curettes, cleaning of the implant surface using a metronidazole gel and a tetracycline hydrochloride solution, and washing with cold sterile physiologic saline solution.	36 months	The cumulative survival rates in the test and control groups were 100% and 87.5%, respectively. The recession index in the control group was significantly lower than that in the test group. PD, CAL, and mBI were lower in the test group than in the control group. Thus, implantoplasty positively influenced oral implant survival.

OFD: open flap debridement, PD: probing depth, CAL: clinical attachment level, Er:YAG: erbium-doped yttrium aluminum garnet laser, mBI: modified sulcus bleeding index, BOP: bleeding on probing.

**Table 5:** List of randomized control studies with and without the use of antimicrobial and supplement during surgical therapy.

Reference Number	Authors	Number of Patients	Intervention	Observation Period	Main Findings
[57]	Hallström H et al. (2017)	39	OFD for surgical treatment of peri-implantitis without any additional therapy. The test group also received AZM on the day of surgery (250 mg × 2) and for four additional days (250 mg per day).	12 months	The adjunctive systemic administration of AZM with OFD did not result in any clinical benefits compared to OFD alone.
[58]	de Waal YC et al. (2013)	30	Resective surgical therapy for peri-implantitis. Patients in the test group underwent decontamination with 0.12% CHX + 0.05% cetylpyridinium chloride.	12 months	No benefits with regard to clinical parameters were observed in the test group compared to those in the control group.
[59]	Teughels W et al. (2021)	21	OFD for surgical treatment of peri-implantitis with oral administration of CS-OSA for 1 year in the test group.	12 months	CS-OSA application led to the stabilization and potential prevention of further bone loss following surgery.

OFD: open flap debridement, mBI: modified bleeding index, OSA: orthosilicic acid, CS-OSA: choline-stabilized orthosilicic acid, CHX: chlorhexidine, AZM: azithromycin.

**Table 6:** List of randomized control studies on the use of regenerative materials during surgical therapy.

Reference Number	Authors	Number of Patients	Intervention		Observation Period	Main Findings
			Test Group	Control Group		
[60]	Wohlfahrt JC et al. (2014)	32	PTGs	-	12 months	No differences in clinical parameters or bone marker levels between the groups.
[61]	Isehed C et al. (2016)	26	Enamel matrix derivative	-	12 months	No statistically significant differences in bone level change and PD difference between the groups.
[62]	Derks J et al. (2022)	138	Cancellous bone granules with10% highly purified porcine collagen	-	12 months	Utilization of the bone substitute material resulted in less pronounced buccal REC but did not lead to improvements in terms of reducing PD and BOP.
[63]	Jepsen K et al. (2016)	63	PTGs	-	12 months	Radiographic defect fill was significantly higher in the test group than in the control group.
[64]	Wohlfahrt JC et al. (2012)	32	PTGs	-	12 months	Radiographic bone fill around implants was significantly higher in the test group than in the control group.
[65]	Emanuel N et al. (2020)	27	Biodegradable prolonged release local doxycycline formulated with β-tricalcium phosphate bone graft	-	12 months	There were statistically significant differences in mean periodontal probing depth, clinical attachment levels, radiographic bone levels, and BOP between the groups. In contrast, there was no statistically significant difference in REC between the groups.
[66]	Solonko M et al. (2022)	49	CM	FGG	12 months	Both treatments groups showed a significant increase in the amount of peri-implant keratinized mucosa width. However, the increase in keratinized mucosa in the control group was significantly greater than that in the test group.
[67]	Schwarz F (2009)	22	NHA	NBM + CM	48 months	NBM + CM application resulted in higher mean PD reduction (NBM + CM: 2.5 ± 0.9 mm vs. NHA: 1.1 ± 0.3 mm) and clinical attachment-level gain (NBM + CM: 2.0 ± 1.0 mm vs. NHA: 0.6 ± 0.5 mm).
[68]	Schwarz F et al. (2008)	22	NHA	NBM + CM	24 months	The application of NBM + CM resulted in higher mean PD reduction (NBM + CM: 2.4 ± 0.8 mm vs. NHA: 1.5 ± 0.6 mm) and mean PD reduction (NBM + CM: 2.0 ± 0.8 mm vs. NHA: 1.0 ± 0.4 mm).
[69]	Schwarz F et al. (2006)	22	NHA	Bovine-derived xenograft in combination with a CM	6 months	Clinically significant PD reductions and CAL gains were observed in both groups.

PD: probing depth, CAL: clinical attachment loss; BOP: bleeding on probing, EMD: enamel matrix derivative, OFD: open flap debridement, mBI: modified sulcus bleeding index, FGG: free gingival graft, REC: mucosal recession, PTGs: porous titanium granules, NHA: nanocrystalline hydroxyapatite, NBM: natural bone mineral, CM: collagen membrane.

## Data Availability

Not applicable.
